# TopoFilter: a MATLAB package for mechanistic model identification in systems biology

**DOI:** 10.1186/s12859-020-3343-y

**Published:** 2020-01-29

**Authors:** Mikołaj Rybiński, Simon Möller, Mikael Sunnåker, Claude Lormeau, Jörg Stelling

**Affiliations:** 1 Department of Biosystems Science and Engineering and SIB Swiss Institute of Bioinformatics, ETH Zurich, Mattenstr. 26, Basel, 4058 Switzerland; 2 ID Scientific IT Services, ETH Zurich, Zurich, 8092 Switzerland; 3 Life Science Zurich Ph.D. program “Systems Biology”, Zurich, 8092 Switzerland

**Keywords:** Ensemble modeling, Bayesian model selection, Topological filtering, Signal transduction

## Abstract

**Background:**

To develop mechanistic dynamic models in systems biology, one often needs to identify all (or minimal) representations of the biological processes that are consistent with experimental data, out of a potentially large set of hypothetical mechanisms. However, a simple enumeration of all alternatives becomes quickly intractable when the number of model parameters grows. Selecting appropriate dynamic models out of a large ensemble of models, taking the uncertainty in our biological knowledge and in the experimental data into account, is therefore a key current problem in systems biology.

**Results:**

The TopoFilter package addresses this problem in a heuristic and automated fashion by implementing the previously described *topological filtering* method for Bayesian model selection. It includes a core heuristic for searching the space of submodels of a parametrized model, coupled with a sampling-based exploration of the parameter space. Recent developments of the method allow to balance exhaustiveness and speed of the model space search, to efficiently re-sample parameters, to parallelize the search, and to use custom scoring functions. We use a theoretical example to motivate these features and then demonstrate TopoFilter’s applicability for a yeast signaling network with more than 250’000 possible model structures.

**Conclusions:**

TopoFilter is a flexible software framework that makes Bayesian model selection and reduction efficient and scalable to network models of a complexity that represents contemporary problems in, for example, cell signaling. TopoFilter is open-source, available under the GPL-3.0 license at https://gitlab.com/csb.ethz/TopoFilter. It includes installation instructions, a quickstart guide, a description of all package options, and multiple examples.

## Background

Uncertainty poses a key challenge for developing predictive models in systems biology [1]. One challenge, parameter inference for systems biology models, has seen important progress in the development and implementation of computational methods that scale to real-world problems [2, 3]. In particular, given that systems biology model parameters are often not uniquely identifiable with the available experimental data, ensemble modeling approaches have gained attention. They represent quantitative uncertainties of biology not by a single parametrization of a model, but by ensembles of parameter values, for applications in areas such as cell signaling [4, 5] and metabolic network analysis [6, 7]. The corresponding methods differ in important details, such as how parameter ensembles are generated; constrained multi-objective optimization [8] and random sampling [9] are possibilities. Bayesian methods such as Approximate Bayesian Computation (ABC) [10–12], a simulation-based method for approximating the Bayesian posterior in parameter space and thereby systematically quantifying uncertainties (in model parameters) [4], are key techniques for model analysis in this context.

These approaches, however, address only one part of the problem in that they assume the underlying network to be uniquely determined. Often also the mechanisms of interactions (the model topology) are uncertain and need to be identified by combining known mechanisms, biological hypotheses, and experimental data. This is a pertinent problem, for instance, for cell signaling studies [13]. If there are few competing hypotheses on mechanisms—leading to few possible model topologies—they can be enumerated and, for example, one can apply ABC to each model topology to select the topology that is most consistent with the data [10, 14–17]. Such Bayesian model selection has been successful for elucidating mechanisms of mammalian epidermal growth factor (EGF) [18] and target of rapamycin (TOR) [19] signaling and of gene regulatory networks in yeast nutrient sensing [20].

With many biological hypotheses, however, the number of possible model topologies explodes in a combinatorial fashion, making enumeration infeasible. To perform model selection in hypothesis spaces with hundreds or thousands of alternatives without full enumeration, three classes of approaches have been proposed: First, it is possible to use simpler, qualitative models to represent alternative biological hypotheses [21, 22], but in this case the quantitative characteristics of the modeled system are not represented. A second option is to combine efficient search in the space of model topologies by formulating a mixed integer optimization problem [23] or by using heuristics to generate candidate topologies [24] with optimization-based parameter estimation. However, this leads to single point estimates for model parameters that do not necessarily reflect the parameter uncertainty, and criteria for model selection that one can use with such point estimates are only justified asymptotically for large numbers of data points, which is rarely the case [25]. The third alternative, ABC for model selection, circumvents these limitations by sampling parameters and topologies (which are again encoded as integers) jointly [10, 15, 16], but with high computational effort and very limited scalability. In particular, these ABC-based methods do not exploit that candidate topologies may be related to each other, preventing a re-use of samples that require costly model simulations between model topologies.

To enable more efficient and scalable Bayesian model selection, we previously proposed a method termed *topological filtering* [26]. While the original method constituted a first assessment of the basic idea, here we describe an implementation in the TopoFilter package that generalizes to a variety of applications in systems and synthetic biology, makes the method directly usable in the form of a well-documented toolbox, and includes new features compared to the version in [26].

## Implementation

### Principle of topological filtering

Biochemical reaction networks, composed of species and reactions that couple them, are the key basis for developing (dynamic) systems biology models. To capture how *l* molecular species interact via *m* reactions, we consider a parametric model $\mathcal {M}(\boldsymbol {p})$ with *d*≥*m* real-valued parameters ***p*** in a bounded parameter space $\mathcal {P}$. Often in systems biology applications, such a parametric model is given in the form of a system of ordinary differential equations (ODEs):

$$ {\frac{d\boldsymbol{x}(t)}{dt}} = \boldsymbol{S}\cdot{\boldsymbol{v}({\boldsymbol{x}(t);\boldsymbol{p}})},\qquad {\boldsymbol{x}(0) =\boldsymbol{x}^{0},} $$

where the state vector ***x***(*t*)≥0 is a time-dependent vector of concentrations of the *l* species. The time-invariant stoichiometric matrix ***S***, which captures how the *l* species interact via the *m* reactions, and the reaction rate laws encoded in the non-negative vector function ***v***, which depends on the *d*≥*m* parameters ***p***, together define the *topology* of the model $\mathcal {M}(\boldsymbol {p})$.

**Fig. 1:**
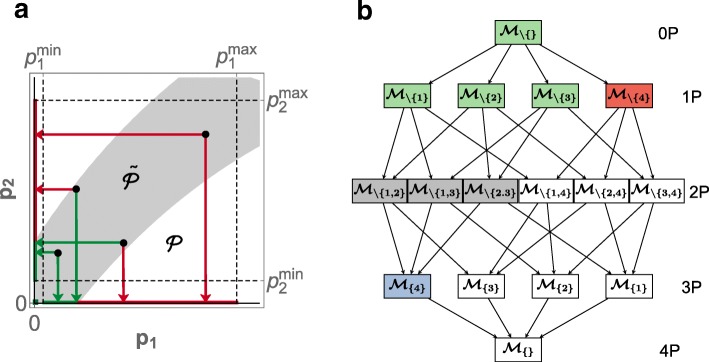
TopoFilter method. **a** Parameter space $\mathcal {P}=\left [p_{1}^{\text {min}},p_{1}^{\text {max}}\right ]\times \left [p_{2}^{\text {min}},p_{2}^{\text {max}}\right ]$ and viable subspace ${\tilde {P}}$ (gray). Sample parameter points (black dots), when projected to zero separately for the two coordinates (arrows), yield viable (green) or non-viable (red) reductions. Colored lines at the axes and a point at the origin denote viable (green) and non-viable (red) lower-dimensional subspaces. **b** Topological filtering step with a rank 1 exhaustive search, for a viable (green) model $\mathcal {M}$ with $\tilde {d}=4$ reducible parameters; $\mathcal {M}_{I}$ ($\mathcal {M}_{\setminus I}$) denotes the model with (without) reducible parameters $I\subseteq \left \{1,\ldots,\tilde {d}\right \}$. For a single viable parameter sample ***p***, all rank 1 parameter reductions (1P) are tested for viability. A union of the three viable 1P reductions skips over the 2P reduction candidates (gray) and goes directly to a single 3P reduction (blue) for viability test. The remaining subspace of models (white) induced by the non-viable 1P reduction (red) is pruned from testing for the current parameter sample. Reductions that have been skipped (gray, white) may still be tested using another parameter sample or in a recursive step

The model generates predictions ***y***(***p***) corresponding to experimental data ***y***^0^ with known measurement errors ***σ***. A scoring function s decides on whether, for a fixed $\boldsymbol {p}, \mathcal {M}(\boldsymbol {p})$ is *viable*—if it describes the data sufficiently well. Correspondingly, we define the *viable subspace* of the parameter space as $\widetilde {\mathcal {P}}=\left \{\boldsymbol {p}\,\left |\mathrm {s} \left ({\boldsymbol {p}; \boldsymbol {y}^{0}}\right)\leq \mathrm {s}^{0}\left ({\boldsymbol {y}^{0}}\right)\right.\right \}$, where s^0^ is a model-independent *viability threshold* (Fig. 1a).

To identify model topologies that are consistent with the data, topological filtering defines a *root model* based on a network that includes the confirmed reactions as well as all hypothetical reactions, and then finds viable reductions of this model. The key idea is to re-formulate model reductions as projections in parameter space (Fig. 1a). A rate law of a biochemical reaction *j*≤*m* is of the form *v*_*j*_(***x***;***p***)=*p*_*j*_·*r*_*j*_(***x***;*p*_*m*+1_,…,*p*_*d*_), with ***x***≥0 the species concentrations and *r*_*j*_ scalar functions corresponding to the concentration-dependent terms [27]. By projecting a *multiplicative kinetic constant* *p*_*j*_ to *p*_*j*_=0, we eliminate reaction *j*. Additional parameters *p*_*m*+1_,…,*p*_*d*_ may be projected to different values. For example, one could project a Michaelis-Menten constant to infinity to remove an enzyme-catalyzed reaction.

For the confirmed reactions in the root model, which represent well-established mechanisms, the associated parameters are non-removable (while they may assume different values, they cannot be projected). For the hypothetical reactions, we consider projections of any subset of their associated $\tilde {d}\leq d$ parameters to given projection values, and each of these subsets defines a *submodel*. Because the values of all *d* parameters are uncertain (parameters of known values are not part of the problem), we not only need to search the topology space of 2^*d*^ candidate submodels (Fig. 1b), but also the parameter space of each submodel. Topological filtering achieves this by filtering candidate reductions and by exploring their lower-dimensional parameter spaces with an efficient sampling algorithm [9].

### TopoFilter algorithm

**Fig. 2:**
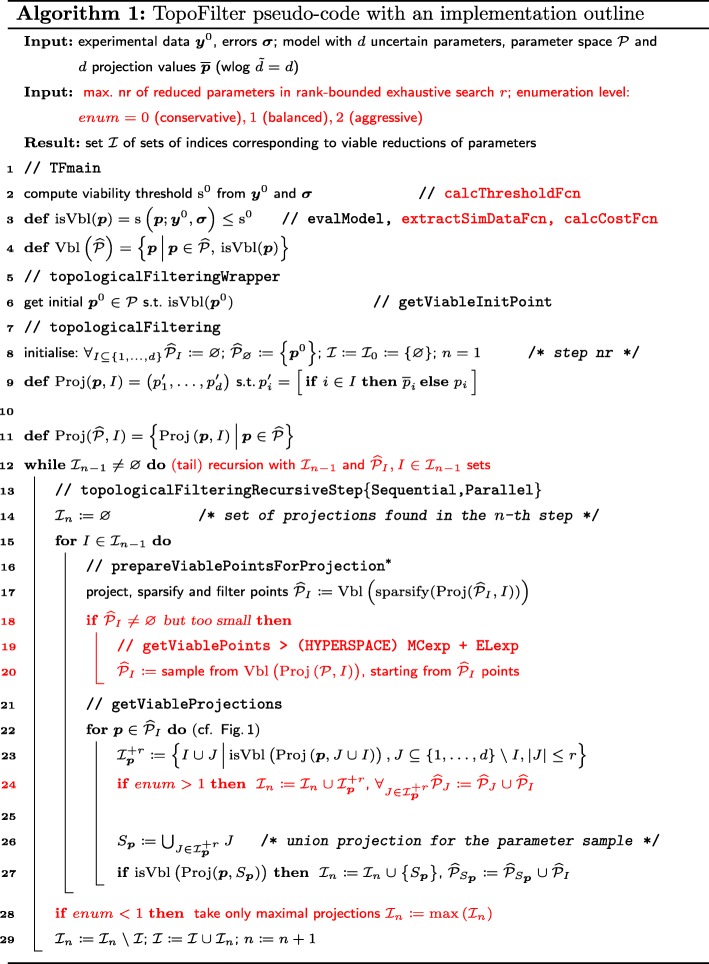
TopoFilter pseudo-code with an implementation outline. Note that, because of an option to re-sample and save viable points for all found projections, preparing points comes actually after the **for**$I\in \mathcal {I}_{n-1}$ loop, in a separate **for**$I\in \mathcal {I}_{n}$ loop, and during initialization. Parts in red highlight differences to the original algorithm [26] such as choice of enumeration level, custom scoring and threshold functions, automatic (tail) recursion, and adaptive resampling from whole (representative) sets of previous samples

Here, we describe the original algorithm for topological filtering in [26], and focus on the new features in the subsequent sections (see also Fig. 2).

Topological filtering starts with the root model (that is, the most complex model without any parameter eliminated, denoted as $\mathcal {M}_{\setminus \{\}}$) and one sample in the viable parameter space for this model (or for short: one viable sample). The initial viable sample can be obtained by standard parameter estimation methods. Starting from such a single viable sample, the original implementation uses a combination of out-of-equilibrium adaptive Monte Carlo (OEAMC) [28] sampling and multiple ellipsoid based sampling (MEBS) [9] to explore the parameter space of the root model. For each sample, the algorithm proceeds by testing if single-parameter projections are viable, thereby identifying possible single-parameter (1P) reductions of the root model (submodels $\mathcal {M}_{\setminus \{1\}}, \mathcal {M}_{\setminus \{2\}}$, and $\mathcal {M}_{\setminus \{3\}}$ in Fig. 1b). Subsequently, a union of 1P reductions resulting from a sample is tested for viability. The 1P and union reduced models with their associated viable parameter samples serve as starting points for the next iterations of the search, until no further reductions are possible. Thus, TopoFilter explores the space of models by reducing viability testing to possibly distant descendants of viable submodels.

### New features

To further develop the method since its first implementation [26], we focused on the scope of applications, the accuracy of the model search, and the computational efficiency. More specifically, the current implementation of TopoFilter includes: (i) customizable scoring functions that enable applications beyond model inference; (ii) simultaneous reductions of several parameters to obtain a viable submodel (see “Results and discussion” section for an example where this feature is important); (iii) adaptive re-sampling to avoid situations in which viable submodels are not detected because the parameter samples from the root model are thinned out during the iterations over the model space; (iv) efficient search heuristics over the model space for cases where we are primarily interested in finding maximal reductions (the most compact submodels that are still consistent with the experimental observations); and (v) more comprehensive options for parallelization, which can avoid redundant searches over model and parameter subspaces. Relevant changes to the algorithm are highlighted in Fig. 2.

#### Customizable scoring functions

By default, we assume uncorrelated and normally distributed errors $\boldsymbol {\mathcal {E}}=\left ({\boldsymbol {y}(\boldsymbol {p})-\boldsymbol {y}^{0}}\right)\sim \mathcal {N}({\boldsymbol {0}, \text {diag}\ {\boldsymbol {\sigma }}})$. The residuals sum-squared error $\Phi =\boldsymbol {\mathcal {E}}^{T}{\text {diag}(\boldsymbol {\sigma })}^{-1}\boldsymbol {\mathcal {E}}$ then are *χ*^2^-distributed. From the distribution of $\boldsymbol {\mathcal {E}}$, the *negative log-likelihood* of the data given the model is *ℓ*=*Φ*/2+*C*, with $C=\ln \left ({\sqrt {(2\pi)^{k}\prod \sigma _{i}}}\right)$ and *k* the number of measured data. TopoFilter uses the default scoring function s≡*ℓ* and a threshold s^0^(***y***^0^) based on quantiles of the *χ*^2^ distribution with *k* degrees of freedom, an upper bound in the standard goodness of fit test for model evaluation. The original scoring function from [26], which relied on a threshold derived as an expected value plus two standard deviations over all data points of the (unknown) true model and its true parameterization, remains available. In addition, TopoFilter supports custom scoring functions, including likelihood-free functions, for example, to enable model-based design in synthetic biology. In such applications, one can score a model according to a desired circuit performance (for example, the ability to adapt to an external signal), without providing experimental data [29].

#### Variable-order projections and parameter coupling

We denote the number of parameters simultaneously tested for projection as the rank *r* of a reduction (relative to the (sub)model the projection is applied to). While the original algorithm supports only reductions of rank *r*=1 and their subsequent unions in one iteration of topological filtering, TopoFilter exhaustively checks reductions up to a given, user-defined rank (and their subsequent unions; Fig. 1b). As illustrated in the theoretical example in the “Results and discussion” section, higher-rank reductions may find valid submodels not detected otherwise because of—not necessarily obvious—couplings between parameters. Moreover, TopoFilter supports a user-defined asymmetric coupling of parameters, for example, to eliminate the associated Michaelis-Menten constants when eliminating the multiplicative kinetic constant for typical enzyme kinetics. Such definitions help increasing the efficiency of model space exploration.

#### Adaptive re-sampling

Originally, the *D* parameter samples in each step of topological filtering carry over from previous steps, and they originate from sampling the root model. In addition to thinning out the samples for higher-order reductions, the distribution of samples in lower-dimensional parameter spaces may not be representative for the corresponding submodels. We therefore provide an option in TopoFilter to obtain new samples adaptively when too few samples are carried over. Re-sampling explores the viable subspace with HYPERSPACE [9], but in contrast to the initial sampling, the OEAMC exploration uses all previously found viable samples to improve MEBS performance. Re-sampling improves discovery of viable submodels but it increases the computational cost. In addition to the model evaluations for parameter space exploration resulting in *D* samples, the number of model evaluations for model space search in each recursive step of TopoFilter is $D\cdot \sum _{i=0}^{r}\binom {\tilde d}{i}$, linear in *D* and in the number of projectable parameters $\tilde d$ for a rank *r*=1 exhaustive search, quadratic for *r*=2, etc. TopoFilter therefore allows the user to control minimal and maximal sample sizes as well as the maximal number of model evaluations per sampling step.

#### Efficient model space exploration

In addition to exploring the model space exhaustively up to a given depth (investigating all reductions up to a given rank for each viable submodel), TopoFilter provides options to speed up the search for higher-order reductions. The algorithm can „jump” heuristically to a union of all viable lower-rank reductions for the currently considered parameter sample and thereby exceed the rank *r* in practice. Each union reduction is then checked against all parameter samples for viability (see the example of how $\mathcal {M}_{\{4\}}$ can be reached in Fig. 1b). TopoFilter may also proceed recursively, where the set of root models for the recursive steps (e.g., $\mathcal {M}_{\{4\}}$, if it is viable for some parameter sample) depends on a user-defined enumeration level to trade off speed and exhaustiveness. TopoFilter supports three enumeration strategies that are selected by their corresponding enumeration levels (in brackets):

- conservative (0): enumerate only maximal viable projections found in a single recursive search step,
- balanced (1): enumerate all viable union projections found for each parameter sample (see Fig. 1b, blue box if found viable), and
- aggressive (2): enumerate all viable projections found, including those found during the initial exhaustive search among low-rank reductions (Fig. 1b, green boxes).

Note that aggressive enumeration is particularly important for model selection, where the trade-off between model complexity and goodness-of-fit needs to be considered.

Finally, TopoFilter implements backtracking as an experimental option. If a reduction of rank *r*>1 is found to be inviable–either for a single parameter sample or for all samples in a iteration of topological filtering–backtracking will test if reductions of lower rank that were ’skipped over’ during model search are viable. For the example in Fig. 1b, if $\mathcal {M}_{\{4\}}$ was inviable, $\mathcal {M}_{\setminus \{1,2\}}, \mathcal {M}_{\setminus \{1,3\}}, \mathcal {M}_{\setminus \{2,3\}}$ would be tested during backtracking.

#### Parallelization

TopoFilter provides options to automatically support parallelization at different levels of the method, allowing adjustments both to the considered case study and to the available hardware. The currently available parallelization levels (in brackets) are:

- (0): Runs are performed sequentially, without parallelization.
- (1): Viability checks and, if required, maximal projections found during viable point preparation are parallelized. This is the least wasteful option compared to sequential runs in terms of computational time because it minimizes the number of redundant model and parameter space searches done in parallel. However, level 1 parallelization has the biggest communication overhead.
- (2): Iterations over root models within a recursive step are carried out in parallel, which is an automation of the parallel strategy in the original method.
- (3): Independent repeats of topological filtering are run in parallel. This option has no additional overhead in terms of computations and communication, and is useful if the number of available parallel cores is small and the method’s results in a particular application have high variance in terms of the model reductions identified.

### Software structure

**Fig. 3:**
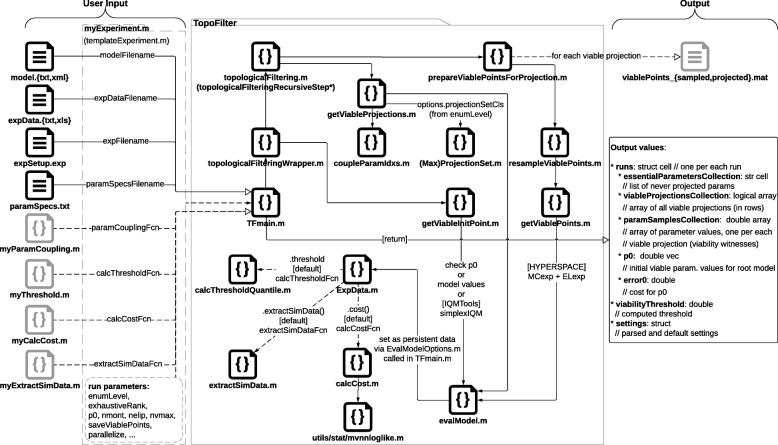
Structure of the TopoFilter package. Call graph diagram of the main TopoFilter function files (middle) with corresponding inputs (left) and outputs (right), denoted with empty arrowheads. Dashed lines and gray boxes indicate optional inputs, call dependencies and outputs. The recommended (optional) own experiment function file can be created from the template experiment function file included in the package or from one of the existing examples

The main inputs, internal dependencies, and outputs of the TopoFilter implementation are summarized in Fig. 3. Mandatory user-defined inputs include the mathematical model, the experimental data (which usually is in the form of time-course data for ODE models), and a specification of the experimental design (e.g., to define time-dependent inputs). Optional inputs allow for the customization of many aspects of TopoFilter’s internal (default) functions, such as the definition of custom scoring functions (see above). Together with parameters for runtime operation (e.g., enumeration level and initial viable parameter vector), these data files and functions are passed to a single main function as the TopoFilter entry point. The main function performs all required computations for topological filtering, returning a single data structure containing the essential outputs, such as each viable projection discovered together with a single witness parameter sample (the memory-consuming sets of all viable parameter samples are optionally written to files on-the-fly; see Fig. 3).

### Implementation details

TopoFilter (v0.3.3) is implemented in MATLAB (MathWorks, Natick, MA) with parallelization support via MATLAB’s Parallel Computing Toolbox. Ordinary differential equation (ODE) models are numerically integrated with SUNDIALS CVODES v2.5 C package [30] via the IQM Tools v1.2.x MATLAB package (IntiQuan GmbH, Basel), which supports SBML [31] and a multi-experiment setup. Sampling uses the HYPERSPACE v1.2.x MATLAB package—an improved version of the implementation described in [9], available at https://gitlab.com/csb.ethz/HYPERSPACE.

For our case studies, all computations were carried out on a homogeneous cluster of Intel ^*Ⓡ*^ Xeon ^*Ⓡ*^ 2.70GHz 24 cores CPUs with 30720KB cache each, running MATLAB R2018b with the Parallel Computing Toolbox.

## Results and discussion

### Case study: target of rapamycin signaling

#### Biological background and study setup

We previously reported applications of topological filtering to models of cell signaling with up to 12 parameters for model selection and up to hundreds of alternative topologies [26]. To test scalability of the improved TopoFilter method for a larger, intracellular signaling pathway model, we focused on target of rapamycin (TOR) signaling. TOR signaling, a pathway responding to the availability of nitrogen sources, is complicated by its connections to other nutrient signaling pathways and because signal transduction involves the control of phosphatases that are hard to analyze experimentally [32]. Dynamic model-based analysis has therefore been instrumental to investigate the pathway’s topology in yeast [33] and mammalian [19] cells, and it suggested complex emergent behaviors in mammalian TOR signaling [34].

**Fig. 4:**
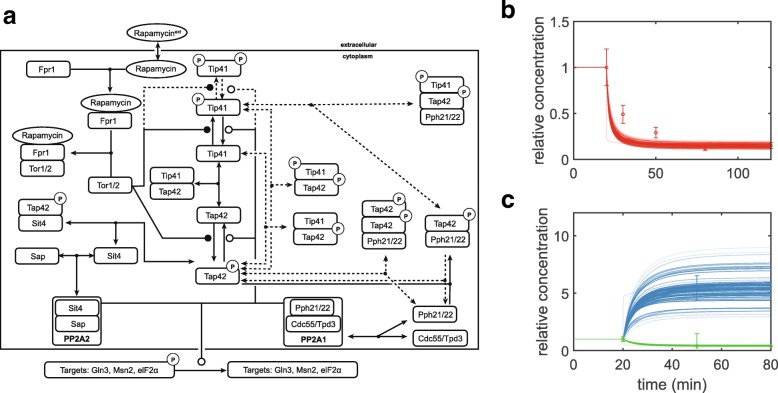
Dynamic model for TOR signaling in budding yeast. **a** Molecular interactions represented in the core model (solid lines) and in hypothetical extensions (dashed lines), adapted from [33]. Nodes represent proteins or protein complexes (boxes; phosphorylation indicated by ’P’) as well as small molecules (ellipses). Arrows indicate reversible complex formation, while filled (open)circles adjacent to transition reactions denote protein phosphorylation (dephosphorylation). **b**, **c** Experimental data (symbols; mean and s.d.) and sample model trajectories (lines) for stimulation of TOR signaling with 500 nM (**b**) and 109 nM (**c**) rapamycin at *t* = 20 min. In (**b**), the abundance of phosphorylated Tap42 protein (red) was measured; in (**c**), complex formations of Tip41 with Tap42 (blue) and of Tap42 with Sit4 (green) were determined. All data are relative to steady-state concentrations prior to rapamycin addition; for details on model structure and experimental data, see [33]. Simulations in (**b**) and (**c**) represent viable parameter samples for a default negative log-likelihood scoring function with a 0.95 quantile as a threshold

For our case study, we use the mass-action kinetics model of the budding yeast target of rapamycin (yTOR) signaling pathway from [33] that includes a core model and several extensions representing hypothetical control mechanisms shown in Fig. 4a. The model captures the core upstream signaling, from TOR complex 1 (TORC1), via the regulatory proteins Tip41 and Tap42, to the heterotrimeric protein phosphatase 2A 1/2 (PP2A1/2) complexes; it includes the drug rapamycin, which binds to TORC1 and inhibits its activity, as an input. As a root model for our analysis, we lumped core model extensions 1–4 to encapsulate a total of 31 state variables and 42 parameters, out of which 18 parameters can be reduced. We used an essential subset of the original experimental data, namely a total of 20 data points for 13 different observable variables, in 3 different experimental conditions (inputs of +0 *μ*M, +109 *μ*M, and +500 *μ*M rapamycin; see example data and simulation results in Fig. 4b-c).

#### Finding the maximal reduction

In this setup with 18 out of 42 parameters being reducible, the core model with 24 parameters is viable, and thus the maximally reduced model. Finding the maximal reduction would require a naïve search to test the whole space of 2^18^=262 144 submodels for viability. To characterize TopoFilter’s performance and accuracy, we therefore first analyzed how the heuristics impact on the speed and probability of finding the maximal reduction.

The data compiled in Table 1 shows that, with the number of model evaluations for the sampling in parameter space varying between 10^2^ and 10^4^, the maximal reduction is always found using the balanced recursive heuristic (enumeration level 1) and the conservative heuristic (enumeration level 0), except for the most greedy search setup with enumeration level 0, rank 1, and the smallest sample size of *n*=10^2^. Time-wise, when searching for the maximal reduction, the conservative enumeration strategy outperforms the balanced enumeration strategy in all cases on a single core (non-parallel), and the performance difference increases with the number of samples *n*. Together, these data indicate that TopoFilter can traverse the model space efficiently and with high reliability.

**Table 1:** TopoFilter performance in finding the maximally reduced TOR signaling model

Enumeration level	Rank *r*	Success rate (%)			Time per run (min)		
		*n*=10^2^	*n*=10^3^	*n*=10^4^	*n*=10^2^	*n*=10^3^	*n*=10^4^
Conservative (0)	1	80	100	100	4	19	154
	2	100	100	100	11	93	733
Balanced (1)	1	100	100	100 ∗	27	178	8’014 ∗
	2	100	100	100	44	215	5’096

Data are averages from five repeated runs on a single worker each, except for the case ∗ with four repeats

#### Covering the model space

**Fig. 5:**
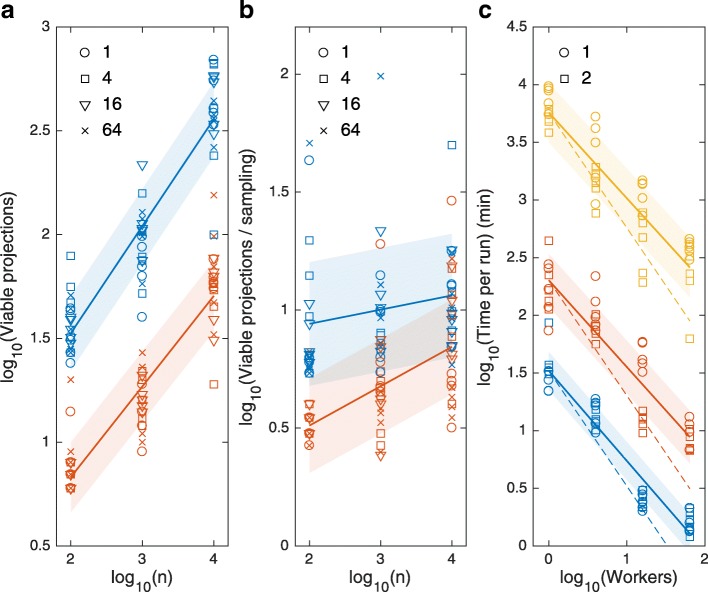
TopoFilter performance for the yeast TOR signaling model. **a** Number of identified viable projections (submodels of the root model) for rank 1 (blue) and 2 (red) exhaustive searches with balanced enumeration strategy, depending on the number of allowed model evaluations in each (re-)sampling step, *n*. Symbols indicate number of workers in parallelized runs (inset). **b** Number of viable projection divided by the number of times the parameter space sampling was carried out when the number of samples left after projecting and filtering was smaller than the user-defined threshold (here: 1/20 of the number of evaluations per sample); symbols are as in (**a**). **c** Run time as a function of number of parallel processes for *n*=10^2^ (blue), *n*=10^3^ (red), and *n*=10^4^ (orange); symbols indicate the enumeration level (inset). The dashed lines give references for a slope of -1. Regression lines (and their standard prediction errors; shaded) were computed for groups per exhaustive search rank *r* (**a**,**b**), or per number of function evaluations *n* (**c**)

Next, to assess how well TopoFilter covers the model space, which is essential for Bayesian model selection, we emphasized simulation studies with the balanced strategy (enumeration level 1). Fig. 5a shows that the total number of discovered projections (submodels) grows with the number of model evaluations as a power function: a higher number of model evaluations allows for a better exploration of the parameter space, as would be expected. In this application, TopoFilter re-samples on average every ca. 3–9 viable projections found, and with each 10-fold increase of the number of model evaluations per sampling, the number of viable projections found per sampling increases by ca. 1.5 fold (Fig. 5b). This implies that re-sampling indeed helps to explore the viable subspace more accurately, and that more often sufficiently many lower-dimensional viable samples are left after a viable projection than without re-sampling.

A comparison of the data for rank 1 and rank 2 exhaustive search in Fig. 5a,b indicates that rank 2 exhaustive search finds fewer projections and (re-)samples more frequently. Although this seems counter-intuitive, the explanation is that in the balanced enumeration strategy (level 1), the total number of all projected parameters in the rank-bounded exhaustive search grows with its rank (projections found in the search are not subject to further recursive steps, only their unions are per parameter sample, see Fig. 2), and bigger projections leave less viable samples after their re-validation. Hence, jumps over levels (ranks) of the model space are bigger for the rank 2 search, but, relative to the number of discovered viable submodels, they imply more frequent re-sampling than for the rank 1 search (Fig. 5b). In addition, for the balanced enumeration strategy, although significantly fewer total projections are found with the rank 2 search (by a factor of 5-10; Fig. 5b), and, hence, fewer iterations are required, the higher (quadratic) cost of testing projections in the rank-bounded exhaustive search steps makes the rank 2 search only slightly faster overall than the rank 1 search (level 1; see Fig. 5b and Table 1). However, in principle, as our theoretical example above demonstrates, the rank 2 exhaustive search allows to find projections that would not be found by eliminating only one parameter at a time.

Finally, for the aggressive enumeration level 2, TopoFilter finds approximately 7– 9·10^4^ viable projections with only *n*=10^2^ average sample size, that is, ca. 25–35% of all 2^18^≈26·10^4^ submodels. The strategy finds the maximal projection quickly, and the vast majority of the computations concerns parts of the model space close to the root model, where previously found small viable reductions from the rank-bounded exhaustive search are systematically extended with single parameters in the following recursive steps (which is not done for enumeration levels less than 2; see Fig. 2).

#### Parallelization and scaling

For the timing data per TopoFilter run in Fig. 5c, it is worth noting that the time required on a single CPU (worker) for *n*=10^4^ samples is approximately 80 h. Hence, TopoFilter can make model selection feasible for a complex practical example such as TOR signaling in reasonable time. The total time per each TopoFilter run decreases with the number of parallel workers linearly (Fig. 5c), showing a good scalability of the method over a parallel computing infrastructure. The most fine-grained parallelization (level 1, in which viability tests are parallelized; see “Implementation” section) allows for significant time improvements with respect to the number of workers, but the average CPU time per worker increases (see increasing gap with respect to the diagonal in Fig. 5c). This is caused by parallelization bottlenecks such as the initial (non-parallel) sampling, and the synchronization after each recursive step, where the method waits for the projections that require the most time-consuming re-sampling.

### Theoretical example

While the case study of TOR signaling indicated performance characteristics of TopoFilter depending on the algorithmic options, it is too complex to systematically identify limitations of the topological filtering heuristic for model selection. We therefore devised a simple, theoretically tractable example network; its analysis motivated in part the method modifications implemented in TopoFilter. In particular, the theoretical example highlights caveats of the heuristic for rank 1 reductions in the exhaustive search as well as the critical nature of the choice of parameter bounds.

Our theoretical example network contains two species with concentrations *x*_1_ and *x*_2_. The first species is only added instantaneously at *t*=0 and degraded with rate *k*_1_*x*_1_(*t*). It acts as a ligand that enhances, with rate *x*_1_(*t*), the production of the second (reporter) species. We assume that the reporter is not present at *t*=0, but that it can be produced at constant rate *k*_2_ and degraded with rate *x*_2_(*t*). This leads to the ODE system:

$$\begin{array}{@{}rcl@{}} \begin{aligned} \frac{dx_{1}(t)}{dt} & = - k_{1} x_{1}(t)\\ \frac{dx_{2}(t)}{dt} & = k_{2}+x_{1}(t)-x_{2}(t), \end{aligned} \end{array} $$

with initial conditions $x_{1}(0)=x_{1}^{0} > 0$ and $x_{2}(0)=x_{2}^{0} = 0$.

With *k*_1_>0, that is, with a degradable ligand, the steady-state of the system is:

$$ x_{1}^{*} = 0, \qquad x_{2}^{*} = k_{2}. $$

However, when we assume a non-degradable ligand (*k*_1_=0), we find that

$$ x_{1}^{*} = x_{1}^{0} > 0, \qquad x_{2}^{*} = x_{1}^{0} + k_{2}. $$

Assume that the correct model is the maximal reduction with *k*_1_=*k*_2_=0. We experimentally observe only the reporter, and only close to the steady-state, such that the model output would be $y_{1} \approx x_{2}^{*}$. For the maximal reduction, $x_{2}^{*}=x_{1}^{0}$, and correspondingly the measurement data for observable *y*_1_ is:

$$ y_{1}^{0} = x_{1}^{0} + \varepsilon, \quad \text{where}\ \varepsilon\sim\mathcal{N}\left({0,{\sigma_{1}}^{2}}\right), $$

that is, the ligand’s initial concentration with a measurement error *ε* that is assumed to be normally distributed with variance equal to *σ*_1_^2^. With TopoFilter’s default negative log-likelihood score and its default 95% quantile threshold *ε*≈1.96*σ*_1_, we have the viability criterion:

$$\left({y_{1}-y_{1}^{0}}\right)^{2}<2{\sigma_{1}}^{2}\cdot\left({1.96\sigma_{1}-\ln({\sqrt{2\pi\sigma_{1}}})}\right), $$

and in terms of parameter samples when parameters are set to 0:

$$\left({y_{1}-y_{1}^{0}}\right) \approx \left\{\begin{array}{ll} 0~+\varepsilon & \quad \text{if}\ k_{1}=k_{2}=0,\\ k_{2}-\varepsilon & \quad \text{if}\ k_{1}=0,\\ x_{1}^{0}+\varepsilon & \quad \text{if}\ k_{2}=0, \end{array}\right. $$

where the approximation becomes more accurate the closer the system is to steady-state at the time point of measurement.

**Fig. 6:**
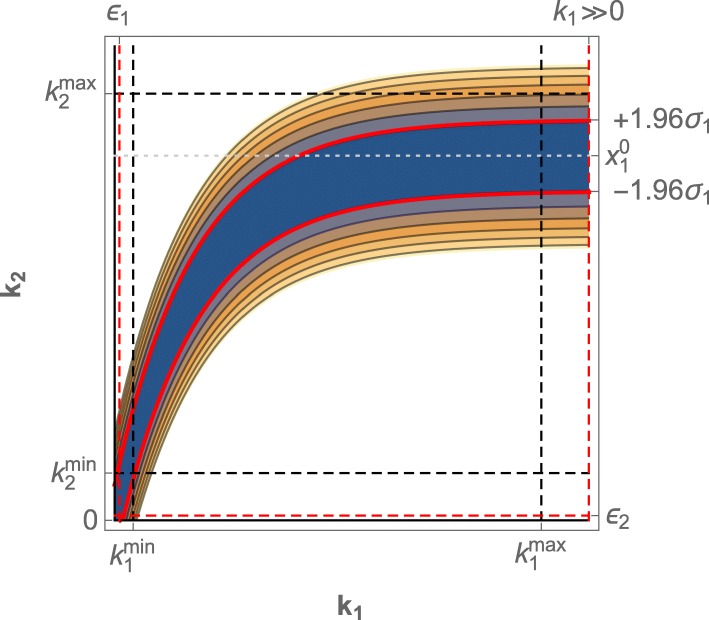
Likelihood and viable space for the theoretical example of a ligand-fluorescent reporter network with multiplicative kinetic constants *k*_1_ and *k*_2_. Bounding box for searched parameter values is given as $\left [{k_{1}^{min},k_{1}^{max}}\right ]\times \left [{k_{2}^{min},k_{2}^{max}}\right ]$ (dashed). The *ε*-projection values for each of the parameters (red dashed, bottom and left) can lead to discovery of the rank 2 reduction via rank 1 reductions (first *k*_1_:=*ε*_1_, then, from viable point $\left (\epsilon _{1},k_{2}^{min}\right), k_{2}\text {:=}\epsilon _{2}$), whereas projection values equal to 0 cannot. With the *k*_1_ projection value greater than $k_{1}^{max}$ (red dashed, right), the viable space, enclosed within a 0.95 quantile of the cost function, is determined only by $k_{2}\in \left ({x_{1}^{0}-1.96\sigma _{1},x_{1}^{0}+1.96\sigma _{1}}\right)$; *k*_1_ can be projected alone to some (high enough) value. The figure was plotted with $\left [{k_{1}^{min},k_{1}^{max}}\right ] \times \left [{k_{2}^{min},k_{2}^{max}}\right ]= \left [{0.0117,0.027}\right ] \times \left [{1.3,11.7}\right ], x_{1}^{0}=10, \sigma _{1}=0.05{\cdot }x_{1}^{0}$

Having fixed the ligand’s initial amount $x_{1}^{0}$ and the lower bound $k_{2}^{min}>0, \sigma _{1}$ can be so small that neither *k*_1_ alone nor *k*_2_ alone can be reduced by projection to 0 as shown in Fig. 6. Hence, when using only the rank 1 exhaustive search, the viable rank 2 reduction of both *k*_1_ and *k*_2_ will not be tested. There are several alternatives to solve or circumvent such issues. One can straightforwardly increase the exhaustive search rank, but this will increase the runtime of a search step. Alternatively, we can choose sufficiently wide bounds for the analyzed region of the parameter space (possibly disregarding known physical bounds)—by decreasing $k_{2}^{min}$ here—but this will decrease sampling accuracy. Finally, if we choose small but positive projection values that approximate the true projection values of 0 (Fig. 6), the caveat is an increased numerical integration time.

For a different scenario, using the same model, the same measurement data, and the same default score function and threshold, consider a different projection value for *k*_1_ at the other end of the range of parameter values, *k*_1_≫0 (Fig. 6). With a *k*_1_ projection value such that the half-life of the ligand is sufficiently small compared to the time point of the measurement *t*^∗^, only a value of the parameter of the constitutive production of the reporter matters for the score. Here, the *k*_2_ value has to be within error bounds of ca. 1.96*σ*_1_ from the $x_{1}^{0}$ value. Given that the upper bound $k_{1}^{max}$ is sufficiently high to allow TopoFilter to find a sample with *k*_2_ value within the error bounds, *k*_1_ can be reduced by projecting to ≫0 value.

Thus, while TopoFilter’s standard setting of testing only single-parameter reductions at a time may prevent finding a maximally reduced model that is consistent with the experimental data, several options exist to prevent or mitigate this potential problem in an application. However, note that these options may lead to increased computational cost or to decreased accuracy.

## Conclusions

The TopoFilter package combines high flexibility in tackling model selection problems in systems and synthetic biology with state-of-the-art, scalable performance. In particular, the user has control over the model space search exhaustiveness and, correspondingly, over the total run-time. TopoFilter’s parameters allow one to choose between search goals: model reduction (maximal viable reduction) and model selection (statistically representative enumeration of viable submodels, the method’s original purpose) are the extremes. The characterization of viable spaces during filtering also enables efficient *post-hoc* uniform sampling for Bayesian computations, which we exploited in prior applications in systems biology [26] and synthetic biology [29] for automated model generation and selection.

We see three main limitations of TopoFilter that could be addressed by future developments: First, parallelization could be extended to the sampling in parameter space, which requires costly model evaluations, in order to improve the computational efficiency and scalability to larger applications. Second, the heuristics for the search in model space could be improved. For example, TopoFilter deals with each parameter sample separately—analyzing the ensemble of viable samples could, for example, help identifying the most promising directions for multi-parameter projections, and thus increase efficiency and accuracy of model space exploration. Finally, TopoFilter currently only provides interfaces for ordinary differential equation (ODE) models, but it could be easily extended to other classes of parametric models. For example, extensions to stochastic network descriptions are particularly straightforward when the dynamics is approximated by so-called moment equations in the form of ODEs [35]. In the future, it could also be interesting to aim for hybrid methods [21] that, for the purpose of model selection, use parameter-free approaches such as logical modeling to constrain the search space for (more detailed) topological filtering a priori as much as possible.

## Availability and requirements

**Project name:** Topological filtering

**Project home page:** https://gitlab.comv/csb.ethz/TopoFilter

**Operating system(s):** Platform independent

**Programming language:** MATLAB

**Other requirements:** MATLAB 2016a or higher, HYPERSPACE 1.2.1 or higher, IQM-tools 1.2.2.2 or higher

**License:** GNU GPLv3

**Any restrictions to use by non-academics:** None

## Data Availability

The datasets generated and/or analyzed during the current study are available in the gitlab repository, https://gitlab.com/csb.ethz/TopoFilter. In particular, the experimental data for the yTOR case study is available in the repository in the ~examples/kuepfer-tor/expData.xls~ file.

## Abbreviations

ABC: Approximate Bayesian computation
EGF: Epidermal growth factor
MEBS: Multiple ellipsoid based sampling
ODE: Ordinary differential equation
OEAMC: Out-of-equilibrium adaptive Monte Carlo sampling
PP2A1/2: Protein phosphatase 2A 1/2 complexes
TOR: Target of rapamycin
TORC1: TOR complex 1
yTOR: Budding yeast target of rapamycin
